# Changes in the Antioxidant Properties of Extra Virgin Olive Oil after Cooking Typical Mediterranean Vegetables

**DOI:** 10.3390/antiox8080246

**Published:** 2019-07-26

**Authors:** Jessica del Pilar Ramírez-Anaya, Ma. Claudia Castañeda-Saucedo, Manuel Olalla-Herrera, Marina Villalón-Mir, Herminia López-García de la Serrana, Cristina Samaniego-Sánchez

**Affiliations:** 1 Department of Nutrition and Bromatology, Pharmacy Faculty UGR, Campus Cartuja s/n, C.P. 10871 Granada, Spain; 2 Department of Nature Sciences, Centro Universitario del Sur (UdeG), Av. Enrique Arreola Silva 883, Ciudad Guzmán C.P. 49000, Jalisco, Mexico; 3 Department of Computational Sciences and Technological Innovation, Centro Universitario del Sur (UdeG), Av. Enrique Arreola Silva 883, Ciudad Guzmán C.P. 49000, Jalisco, México

**Keywords:** fry, boil, phenolic compounds, antioxidant capacity, extra virgin olive oil, mediterranean vegetables

## Abstract

Extra virgin olive oil (EVOO), water, and a water/oil mixture (W/O) were used for frying, boiling and sautéeing Mediterranean vegetables (potato, pumpkin, tomato and eggplant). Differences in antioxidant capacity (AC) (2,2-diphenyl-1-picrylhydrazyl (DPPH), ferric iron (FRAP), 2,2-azinobis-(3-ethylbensothiazoline)-6-sulphonic acid (ABTS)), total phenolic content (TPC) and individual phenols (high-performance liquid chromatography (HPLC)) in unused and used EVOO and water were determined. The water used to boil tomatoes showed the highest TPC value, whilst the lowest was found in the EVOO from the W/O used for boiling potatoes. After processing, the concentrations of phenols exclusive to EVOO diminished to different extents. There was a greater transfer of phenols from the vegetable to the oil when eggplant, tomato and pumpkin were cooked. W/O boiling enriched the water for most of the phenols analysed, such as chlorogenic acid and phenols exclusive to EVOO. The values of AC decreased or were maintained when fresh oil was used to cook the vegetables (raw > frying > sautéing > boiling). The water fraction was enriched in 6-hydroxy-2,5,7,8–tetramethyl-chroman-2-carboxylic acid (Trolox) equivalents following boiling, though to a greater extent when EVOO was added. Phenolic content and AC of EVOO decreased after cooking Mediterranean diet vegetables. Further, water was enriched after the boiling processes, particularly when oil was included.

## 1. Introduction

The Mediterranean diet is characterised by high consumption of extra virgin olive oil (EVOO), either as a dressing or in cooking [1,2,3]. EVOO is a source of phenolic compounds with a powerful antioxidant activity that includes hydroxytyrosol, tyrosol and secoiridoid derivatives [4,5]. The supply of phenols in the diet complements the activity of endogenous antioxidant defences. In turn, the ingestion of these compounds has been linked to important effects on public health diseases. For instance, as molecules that promote healthy ageing and as prophylactic agents against physiopathological conditions such as colorectal, prostate and breast cancer, chronic-degenerative diseases, and metabolic syndrome [6,7,8,9,10,11]. Vegetables frequently consumed in Mediterranean populations, such as eggplant (aubergine), potato, tomato and pumpkin, are often cooked in different ways using EVOO. Cooking helps to guarantee safety and produce favourable physical-chemical alterations, which determine the nutritional quality and acceptability [1,12,13,14]. The effects of domestic processing on total and individual phenol concentrations and the antioxidant capacity of foods (AC) have been studied elsewhere, finding either increases or decreases in processed vegetables [1,15,16,17]. In this way, a specific phenolic and antioxidant activity profile have been developed for each cooked vegetable that results from the interaction of the raw vegetable’s characteristics and the cooking techniques tested [12]. At the same time, gains have been reported in cooking water during soup elaboration with extra virgin olive oil and different vegetables [4,18]. A wide range of changes in phenolic content has been found in EVOO. These include degradation and loss through oxidation, hydrolysis and polymerisation reactions [19] which are accentuated during heating in the presence of foods such as meat or potatoes [18]. Furthermore, the incorporation of bioactive compounds from processed vegetables has been documented, as in the case of naringenin, ferulic acid, and quercetin during the production of tomato *sofrito* sauce [1]. The aforementioned changes depend on the chemical structure and the magnitude of antioxidant activity of the compounds [20,21]. Approaches to generalise variations in phenolic antioxidants have been suggested, to determine optimal cooking processes. One example is the functional mathematical index [22], which predicts alterations in specific nutritional and bioactive components in olive oils, green tea and potatoes.

This study aims to determine changes in the phenolic content and profile, and in the antioxidant capacity of extra virgin olive oil, water and their combinations when used to cook four typical Mediterranean vegetables using frequently used domestic techniques.

## 2. Materials and Methods

### 2.1. Chemicals

For extraction, methanol, ethanol, acetone and hydrochloric acid (37%) were supplied by Panreac (Barcelona, Spain). Folin-Ciocalteu phenol reagent purchased from Merck (Darmstadt, Germany) was used for total phenolic content quantification. The reagents used to measure the antioxidant activity, 2,2-azinobis-(3-ethylbensothiazoline)-6-sulphonic acid (ABTS), 2,2-diphenyl-1-picrylhydrazyl (DPPH) and 6-hydroxy-2,5,7,8–tetramethyl-chroman-2-carboxylic acid (Trolox) standard were acquired from Sigma-Aldrich (Milan, Italy), while 2,4,6-tri(2–pyridyl)-s-triazine (TPTZ) for the ferric iron (FRAP) method were from Fluka Chemicals (Buchs, Switzerland). Sodium acetate 3-hydrate, anhydrous sodium carbonate, acetic acid glacial, ferric chloride 6-hydrate, were also from Panreac (Barcelona, Spain). During high-performance liquid chromatography (HPLC) analysis, mobile phases used (methanol and orthophosphoric acid) were from the same provider of extraction reagents and solvents, and ultrapure water was obtained with a Milli-Q system (Millipore, Bedford, MA, USA). Standards were purchased from Sigma-Aldrich (Milan, Italy) in the case of pinoresinol, quercetin, luteolin, apigenin and gallic, p-hydroxybenzoic, p-hydroxyphenylacetic and vanillic acids, from Fluka Chemicals (Buchs, Switzerland) the 3,4-dihydroxybenzoic, chlorogenic, caffeic, syringic, p-coumaric, o-coumaric and ferulic acids, tyrosol, o-vanillin and oleuropein from Extrasynthèse (Lyon, France), and rutin from HWI Analytik GMBH pharma solutions (Ruelzheim, Germany). The Organic Chemistry laboratory in the Faculty of Pharmacy of Granada University synthesized hydroxytyrosol standard. All chemicals were analytical reagent grade.

### 2.2. Foodstuffs

EVOO in 5 L plastic bottles (Seville, Spain) and fresh potato (*Solanum tuberosum*), pumpkin (*Cucurbita moschata*), tomato (*Lycopersicum esculentum*), and eggplant (*Solanum melongena*), were acquired in commercial establishments in Granada (Spain).

### 2.3. Cooking Conditions

Deep frying and sautéing using EVOO, boiling in water and boiling in a water/EVOO mixture (W/O) were chosen as domestic cooking techniques to study the changes in phenolic compounds and AC of the heat transfer media. Distilled water was included in boiling assays, and unused oil and water were used as a control. The temperature and proportion (w/w) of heat transfer medium and foodstuff were: 180 °C and 5:1 for deep-frying, 80–100 °C and 0.5:1 for sautéing, and 100 °C and 5:1 for boiling. For boiling W/O, the proportions of water, oil and foodstuff were 4.5:0.5:1. Each test was carried out using 120 g of vegetable previously washed and dried. The potatoes and pumpkins were peeled, and the seeds removed from the pumpkins and the tomatoes, and separately cut into one centimeter cubes. The pieces obtained from three units of each vegetable were mixed to avoid unnecessary variations. In all cases, the processing times were held for 10 min at the fixed temperatures. The heat transfer medium was recovered after draining the vegetables for 5 min; additionally, the W/O medium was separated in a decantation device. Each sample was stored under cool and dark conditions until extraction of the phenolic compounds. The assays of the four cooking techniques with each vegetable were carried out in triplicate.

### 2.4. Extraction Conditions

The extracts were obtained by combining 10 g of oil with 10 mL of 80% methanol (1:1 *w/v*) and shaking for 60 min. The mixture was then centrifuged at 6800 g for 15 min. After recovery of the polar fraction, the procedure was repeated. Volume was adjusted to 25 mL with 80% methanol [23]. The boiling water samples were centrifuged under the same conditions to eliminate solid particles [24]. The extracts and boiling water were stored at −40 °C for no longer than two months.

### 2.5. Phenol Contents

#### 2.5.1. Total Phenolic Content (TPC)

The total phenolic content of used water or methanolic oil extract was determined in triplicate using the Folin-Ciocalteu colorimetric method [25] modified in our laboratory [26]. Measuring the absorbance at 700 nm using a Boeco S-22 ultraviolet-visible (UV-VIS) spectrophotometer (Hamburg, Germany). The TPC was calculated through a calibration curve of gallic acid (0.5–7.5 μg/mL) (*R =* 0.999) and expressed as mg gallic acid equivalents (GAE)/g.

#### 2.5.2. Individual Phenol Content by High-Performance Liquid Chromatography (HPLC)

A Varian Pro Star HPLC system equipped with an autosampler, binary pump, UV detector and System Control-Konik software was used for chromatographic determination in triplicate. The chromatographic separation was performed in a Tracer Extrasil ODS25 µm, 15 × 0.4 cm (Teknokroma, Barcelona, Spain) column, following a previously published method with some modifications [27] with a flow rate of 1.0 mL/min and an injection volume of 5 µl. The mobile phases were acidified water (0.01 M phosphoric acid) and methanol as solvent A and B, respectively. The following multi-step binary gradient was used: 0.0 min (A:B 95/5), 25 min (A:B 60/40), 15 min (A:B: 50/50), 5 min (A:B 30/70), 10 min (A:B 20/80), 5 min (A:B 0/100). Measurements were taken at 280 nm and peaks were identified by the external standard method according to retention times of phenols mixed in a standard solution (0.5–250 µg/mL for each phenol). The stock standard was added to problem samples to verify the increase in the area of the chromatographic peak. Results were expressed as µg/g.

### 2.6. Antioxidant Capacity (AC)

AC of the samples was measured by three methods: The DPPH scavenging assay [28], that based on the reducing ability of FRAP [29], and the antioxidant equivalent capacity assay based on the reduction of the radical cation ABTS [30]. The absorbance of the samples was measured at 515, 593 and 734 nm, respectively, in a BMG Labtech FLUOstar Omega plate reader (Offenburg, Germany) using the Omega Control program and MARS Data Analysis Software. The antioxidant activity was calculated from the change in absorbance after sample addition using the regression equations from different calibration curves ranges depending on the method. The results were expressed as µm Trolox equivalents (TE)/g. Antioxidant capacity was determined in triplicate.

### 2.7. Statistical Analysis

Statistical analysis was carried out using the statistical package SPSS for Windows v. 25 (IBM Corporation, New York, United States). The data obtained in oil and water recovered from the different cooking experiments were compared with unused cooking media using analysis of variance (ANOVA) and Tukey’s test. Subsequently, simple linear correlation analysis was used. Differences were considered significant when the *p* value was <0.05.

## 3. Results and Discussion

### 3.1. Total Phenol Contents in Oil and Water Fractions

Table 1, Table 2, Table 3 and Table 4 show the values obtained from the analysis of the water and oil fractions after the application of the four cooking techniques to the vegetables. Data from the unused water were omitted due to measurements of null TPC. The highest TPC value for the two fractions was found in the water used to boil tomatoes, whereas the lowest corresponded to the EVOO recovered from the W/O used to boil potatoes. The TPC values measured in the heat transfer medium were lower than those previously reported in processed and raw potato, eggplant, tomato and pumpkin cubes [12].

In the EVOO, maximum concentrations were found in the fresh oil used for boiling pumpkin. In water, the minimum value was measured after boiling the same vegetable. TPC decreased in the processed EVOO. Furthermore, whilst the water was enriched after boiling, the phenol content of the recovered oil and water did not exceed that of the fresh oil. Previous observations showed a progressive reduction in the concentration of polyphenols of EVOO during prolonged heating in a microwave oven and during frying at high temperatures. This reduction was attributed to thermal oxidation reactions, polymerisation and hydrolysis, or to covalent linking between oxidated phenols and proteins or aminoacids [31,32,33]. Additionally, leaching from EVOO to water during boiling has been reported as a cause of TPC reduction, with higher overall losses from the oil when vegetables were included [1,18,34]. This transference accounted for the rise in TPC of cooking water, with most of the oil’s compounds being found in this fraction. When present during boiling, vegetables also contribute to the leaching of substances that are released from disrupted cell walls and subcellular compartments [35,36]. This has been evidenced in boiled beans [37], cauliflowers [38], and green peppers [39].

### 3.2. Individual Phenol Content of the Oil and Water Fractions by Liquid Chromatography (HPLC)

The concentrations of phenolic compounds exclusive to EVOO [40,41] uch as hydroxytyrosol, tyrosol, oleuropein and pinoresinol, were lower in processed oil than in raw EVOO, as can be seen in Table 1, Table 2, Table 3 and Table 4 (null contents of unused water were omitted). A significant tendency to decrease following cooking was evident after application of most of the techniques to all of the included vegetables. However, these phenols did not show similar behavior. Hydroxytyrosol and tyrosol presented major content losses, down to complete elimination after sautéeing and boiling the vegetables in W/O. On the other hand, a reduction, conservation and even an increase in pinoresinol content was seen. These results agree partially with previous studies on the sterilization of W/O and water/saline mixtures [42,43,44,45,46]. Authors have previously explained these changes as resulting from a number of different causes. One of these relates to the same thermal degradation reactions mentioned in Section 3.1 in relation to TPC [47], that leads to lower survival of polyphenols, such as oleuropein and hydroxytyrosol, in oil when frying potatoes in olive oil enriched with olive leaf extracts [48,49]. As also mentioned in Section 3.1, as a result of the leaching phenomena from EVOO either to the vegetable or to the water, concentrations in oil are diminished after frying, sautéeing and boiling [1,12]. In our study, dissolution in cooking water was confirmed as the phenol compounds are only quantified in liquid after boiling it in W/O mixtures, with tyrosol being the compound with the highest transfer rate. The high solubility of hydroxytyrosol, tyrosol, oleuropein and pinoresinol has been previously established [5,50]. Oleuropein is known to be the most hydrophilic at low temperatures [11,51,52]. In the present study, this favors its migration from EVOO into the cooking water. In contrast, pinoresinol is the most hydrophobic compound [53]. This explains why the trend towards its loss in oil was less marked or even contrary to that of the other three phenols exclusive to EVOO. It also explains the lower magnitudes (0% to 372.22%) with which the concentrations of pinoresinol was present in cooking water relative to those found in unused EVOO. This is compared to the percentages calculated for hydroxytyrosol (603.54% to 2286.2%), tyrosol (372.07% to 728.40%) and oleuropein (84.25% to 1055.97%). Equally high increases in water have been reported in other studies using a simulation of cooking processes involving pressure cooking with W/O [54,55]. In this case, tyrosol concentration in the aqueous phase increased by 1350%, to a greater extent that in original oil. The author stated that following total hydrolysis of the combined forms of tyrosol and hydroxytyrosol in the oil phase, the free form was transferred into water. This resulted in a higher total overall in the cooking water than in the original oil.

Other phenolic compounds such as rutin and chlorogenic acid were not found in the profile of raw EVOO. Following the cooking process of vegetables, rutin remained absent in the oil but chlorogenic was found to be present when tomato and eggplant were fried (Table 1, Table 2, Table 3 and Table 4). During all cooking experiments apart from those with potato, transference from the vegetable also lead to increases in the concentrations of compounds already present in the fresh oil, such as gallic, dihydroxybenzoic, hydroxybenzoic and hydroxyphenylacetic acids, as well as luteolin, apigenin and vanillic acid. The decrease in phenols is, therefore, an anticipated trend given the reasons stated above. However, transference of phenols from the vegetable to the oil was unpredicted. The greater polarity of phenols favours their migration from scarcely polar interfaces (EVOO) towards polar interfaces (the water of the cooking medium or that of the vegetable). This explains why movement towards a polar medium does not occur spontaneously [56,57]. Although phenols (including chlorogenic acid) exhibit some activity in water-oil interfaces [58], this activity is not strong enough because they contain functional groups such as the carboxylic acids, which also make them highly hydrosoluble. However, the extraction and retention of phenolic compounds moving in the opposite direction have also been observed in experimental models of phenol extraction with conventional oils, with EVOO being one of the oils with the highest extraction yield [59]. The magnitude of this phenomenon depends on the refining process and the presence of amphiphilic compounds or compounds with alcohol or acid groups in the oil (i.e., free fatty acids, partial glycerides, phospholipids, phytosterols, etc.) [60]. In raw vegetable oils, these molecules give rise to the formation of lamellar structures consisting of fine alternating layers of water and oil separated by surfactant layers such as suitably oriented phospholipids and sterols [61]. The formation of these structures could explain the increase in phenol concentration observed in some of the oil samples examined in the present study.

Given the zero phenol content of pure water, the cooking medium in both boiling techniques was enriched with most of the phenols analysed. For this reason, it is strongly recommended to use less water, reduce cooking time and to consume the water used during boiling in order to obtain the benefits of the compounds found in different foods [39]. The number of phenols transferred and the magnitude of these cases was higher when oil was included during boiling. Examination of the four phenols exclusive to EVOO that were detected in the water of the W/O mixture (Table 1, Table 2, Table 3 and Table 4), together with the quantification of chlorogenic acid, prove the simultaneous contribution of the vegetable to the phenolic profile of the cooking water and that of the oil.

### 3.3. Antioxidant Capacity (AC)

The AC of water and EVOO are summarized in Table 1, Table 2, Table 3 and Table 4. In the EVOO, the values of fresh oil decreased or were maintained after being used to cook the vegetables (DPPH, ABTS, FRAP). Exceptions were found in the oil used for frying, with significant increases (*p* < 0.05) after frying potato (ABTS), pumpkin (FRAP) and tomato (DPPH, ABTS, FRAP). Further processes also proved exceptions, specifically sautéing eggplants (ABTS) and boiling potatoes in W/O (FRAP). The cooking techniques were classified in decreasing order according to the AC measured in the recovered EVOO. The order was as follows: raw > deep frying > sautéing > boiling. This pattern was more consistent with the results obtained by DPPH (from potato, eggplant and pumpkin) than with those of ABTS (pumpkin) and FRAP (eggplant). Given the establishment of a significant correlation between the phenol concentrations and the AC measured by FRAP (R = 0.76), DPPH (*R* = 0.47) and ABTS (*R* = 0.31), it is concluded that the reduction in AC of EVOO is associated with the loss of phenols [33,34,35,36,37].

On the other hand, the water fraction recovered from the boiling tests was enriched in Trolox equivalents. The AC of the boiling water was found to have the lowest values when oil was not added (DPPH, ABTS and FRAP). Further, AC was negligible (0 µmol ET/g) after boiling pumpkins (DPPH, FRAP), tomatoes and potatoes (FRAP), and eggplants (DPPH). Migration of high amounts of substances into the cooking medium, with a parallel increase in AC, has been observed after boiling purple waxy corn [62] and bamboo shoots [63].

### 3.4. Correlations

The correlation between the AC quantified by the three methods (FRAP, DPPH and ABTS) and the TPC was significant (*p* < 0.05) and positive in the case of unused and processed oil (*R* = 0.76, 0.47 and 0.31, respectively). In the case of water, this was only true when AC was measured through the DPPH method (*R* = 0.77). The correlation between the AC measured by DPPH and ABTS, and the TPC in extra virgin olive oil has been reported before, although the values of the correlations reported previously were higher than those obtained in the present study [64,65]. The low correlation between TPC and AC as measured by the ABTS method can be explained by the original use of this technique in the analysis of hydrosoluble compounds. This contrasts to the present study which examined molecules of varying polarity [66].

Following examination of the correlations of individual phenols with the AC of the recovered EVOO, high correlation coefficients were found with pinoresinol when measured using FRAP (*R* = 0.690) and DPPH (*R* = 0.642). This was also the case with *o*-coumaric acid (*R* = 0.726, FRAP), and with caffeic acid and hydroxytyrosol according to DPPH (*R* = 0.690 and 0.778, respectively). There were few significant correlations with AC when measured by ABTS. Those that were significant were generally weak. The coefficients in cooking water were high when correlated AC with phenols other than those exclusive to EVOO, such as *p*-coumaric (*R* = 0.740, FRAP and DPPH), syringic (*R* = 0.654), chlorogenic (*R* = 0.876), hydroxybenzoic (*R* = 0.800) and dihydroxybenzoic (*R* = 0.664) acids, and with the values obtained by DPPH in the cases of apigenin (*R* = 0.654), caffeic (*R* = 0.627) and hydroxyphenylacetic acids (*R* = 0.714). The AC of boiling water as measured by ABTS produced the highest correlations of the whole study in the cases of *p*-coumaric (*R* = 0.916), syringic (*R* = 0.628), chlorogenic (*R* = 0.884), hydroxybenzoic (*R* = 0.895) and dihydroxybenzoic (*R* = 0.616) acids. The ability of the various phenolic compounds to interact with the radicals used in the ABTS, DPPH and FRAP methods was found to vary according to structural factors belonging to each compound. Such factors include the presence or lack of glycosidic moieties and catechol groups in the polyphenol, the glycosylation site, and the number and position of free and esterified hydroxyls, amongst others [67]. This property is heightened in the presence of a catechol group and multiple hydroxyl groups, as is the case of hydroxytyrosol, which has an extremely high antioxidant capacity. In addition, the differentiated response observed with the ABTS method caused by the different nature of the two heat transfer mediums studied, has been established as a variation factor between the results obtained by the various antioxidant methods. This differentiated response is needed as the outcomes produced can vary substantially due to differences in reaction mechanisms and the solvents involved. In the case of olive oil, the complexity of the food matrix can be a factor accentuating these differences [64,68].

## 4. Conclusions

Changes in the general antioxidant properties of extra virgin olive oil after cooking typical Mediterranean vegetables were demonstrated following the use of different culinary techniques. Phenolic content and antioxidant capacity of the extra virgin olive oil decreased after cooking Mediterranean diet vegetables, regardless of the technique used. It was found that during cooking in domestic conditions, contact between polar (vegetables or cooking water) and non-polar (oil) fractions was favored. In this way, the migration of phenols between the oil and the vegetables or the cooking water was facilitated in both directions. The individual phenolic compounds showed different migration trends between vegetable and oil, leading to changes in the profile of the fresh heat transfer medium as a result of loss or inclusion of phenols. On the other hand, when water was included it was enriched after the boiling processes, particularly when oil was added to the cooking medium. Based on these findings, it is recommended that all ingredients included during food preparation be accurately dosed to ensure total consumption without eliminating the oil or water used for cooking. The former will guarantee a complete phenolic profile and greater bioavailability in the diet. However, the proportion of oil relative to the food is so high in deep frying that, even when drained, those foods impregnated with a higher quantity of oil present a calorie amount that means their frequent consumption cannot be recommended. That being said, cooking techniques with the lowest proportion of oil ensure the most efficient use of EVOO. Through other less conventional methods such as microwave frying, the sensorial characteristics of fried food can be achieved whilst optimally controlling the negative effects on phenolic antioxidants and excluding the deleterious rise in calorie density. It is important to characterize the changes generated by conventional methods, as much as by emergent ones, on the functional compounds and lipid profiles of oils with high biological value such as EVOO. This has the purpose of deepening knowledge of the impact of elaboration methods on the health benefits associated with the Mediterranean diet.

## Figures and Tables

**Table 1:** Phenolic concentration and antioxidant capacity of extra virgin olive oil (EVOO) and water used during the cooking of potatoes according to four techniques.

Domestic Cooking technique	Unused	Deep Fried	Sautéed	Boiled (W/O)	Boiled	Boiled (W/O)
Heat Transfer Medium	Extra Virgin Olive Oil				Water	
**Total Phenol Content (mg GAE/g)**						
Total phenols	0.241 ± 0.020 ^e^	0.21 ± 0.02 ^d^	0.095 ± 0.003 ^c^	0.023 ± 0.001 ^ab^	0.039 ± 0.004 ^b^	0.081 ± 0.003 ^c^
**Individual Phenol Content (µg/g)**						
Gallic acid	0.012 ± 0.001 ^a^	Traces	nd	0.0130 ± 0.0002 ^a^	0.091 ± 0.002 ^b^	Traces
Hydroxytyrosol	0.125 ± 0.002 ^a^	0.1250 ± 0.0001 ^a^	0.082 ± 0.002 ^a^	nd	nd	2.014 ± 0.160 ^b^
3,4-dihydroxybenzoic acid	nd	nd	nd	nd	0.759 ± 0.001 ^a^	0.759 ± 0.020 ^a^
Tyrosol	0.114 ± 0.010 ^b^	0.1180 ± 0.0001 ^b^	0.066 ± 0.002 ^a^	nd	nd	0.857 ± 0.080 ^c^
*p*-hydroxyphenylacetic acid	nd	nd	nd	nd	nd	1.646 ± 0.090
*p*-hydroxybenzoic acid	nd	nd	nd	nd	0.324 ± 0.020 ^b^	0.292 ± 0.020 ^a^
Chlorogenic acid	nd	nd	nd	nd	1.136 ± 0.080 ^b^	0.818 ± 0.040 ^a^
Vanillic acid	nd	nd	nd	nd	0.618 ± 0.030 ^a^	0.749 ± 0.060 ^b^
Caffeic acid	0.011 ± 0.001 ^a^	Traces	Traces	nd	nd	0.089 ± 0.010 ^b^
Syringic acid	0.019 ± 0.001 ^b^	0.019 ± 0.001 ^b^	0.016 ± 0.001 ^a^	nd	nd	nd
*p*-coumaric acid	0.050 ± 0.001 ^a^	0.081 ± 0.002 ^c^	0.122 ± 0.010 ^d^	0.071 ± 0.010 ^b^	nd	0.351 ± 0.020 ^e^
*o*-vanillin	0.368 ± 0.020 ^b^	0.418 ± 0.020 ^c^	0.108 ± 0.010 ^a^	nd	nd	1.703 ± 0.10 ^d^
*o*-coumaric acid	0.022 ± 0.001 ^b^	0.01700 ± 0.00003 ^ab^	0.0130 ± 0.0004 ^a^	0.0180 ± 0.0004 ^ab^	0.633 ± 0.020 ^d^	0.143 ± 0.010 ^c^
Oleuropein	0.429 ± 0.030 ^c^	0.129 ± 0.002 ^a^	0.335 ± 0.010 ^b^	0.149 ± 0.010 ^a^	nd	1.385 ± 0.110 ^d^
Pinoresinol	0.454 ± 0.020 ^c^	0.478 ± 0.040 ^c^	0.252 ± 0.020 ^b^	0.103 ± 0.010 ^a^	nd	1.749 ± 0.150 ^d^
Luteolin	0.101 ± 0.002 ^b^	0.101 ± 0.001 ^b^	0.091 ± 0.010 ^a^	nd	nd	nd
Apigenin	0.142 ± 0.010 ^a^	0.14 ± 0.01 ^a^	nd	0.432 ± 0.040 ^b^	nd	0.096 ± 0.010 ^c^
Rutin	nd	nd	nd	nd	nd	nd
**Antioxidant Capacity (µmol TE/g)**						
DPPH	1.324 ± 0.020 ^e^	1.295 ± 0.050 ^e^	0.516 ± 0.030 ^d^	0.080 ± 0.004 ^ab^	0.103 ± 0.040 ^b^	0.212 ± 0.010 ^c^
FRAP	1.439 ± 0.110 ^b^	1.505 ± 0.010 ^bc^	0.602 ± 0.050 ^a^	2.062 ± 0.070 ^d^	nd	nd
ABTS	1.172 ± 0.070 ^c^	5.371 ± 0.350 ^e^	0.889 ± 0.020 ^b^	0.499 ± 0.020 ^a^	0.621 ± 0.040 ^a^	1.689 ± 0.030 ^d^

Nd = not detected, W/O = Water/oil mixture. ^a–e^ Means with different letters within the same line are significantly different (*p* < 0.05). DPPH: 2,2-diphenyl-1-picrylhydrazyl; FRAP: Ferric reducing ability of plasma; ABTS: 2,2′-azino-bis(3-ethylbenzothiazoline-6-sulphonic acid.

**Table 2:** Phenolic concentration and antioxidant capacity of EVOO and water used during the cooking of eggplants according to four techniques.

Domestic Cooking Technique	Unused	Deep Fried	Sautéed	Boiled (W/O)	Boiled	Boiled (W/O)
Heat Transfer Medium	Extra Virgin Olive Oil				Water	
**Total Phenol Content (mg GAE/g)**						
Total phenols	0.358 ± 0.003 ^e^	0.208 ± 0.010 ^cd^	0.049 ± 0.001 ^ab^	0.0450 ± 0.0001 ^ab^	0.196 ± 0.010 ^cd^	0.141 ± 0.010 ^bc^
**Individual Phenol Content (µg/g)**						
Gallic acid	Traces	0.0110 ± 0.0002	nd	nd	nd	nd
Hydroxytyrosol	0.141 ± 0.004 ^c^	0.112 ± 0.002 ^b^	0.082 ± 0.002 ^a^	0.084 ± 0.002 ^a^	nd	0.992 ± 0.010 ^d^
3,4-dihydroxybenzoic acid	nd	0.081 ± 0.002 ^a^	0.067 ± 0.002 ^a^	nd	1.021 ± 0.020 ^c^	0.982 ± 0.040 ^b^
Tyrosol	0.099 ± 0.004 ^b^	0.093 ± 0.004 ^b^	0.066 ± 0.002 ^a^	0.065 ± 0.001 ^a^	nd	0.743 ± 0.020 ^c^
*p*-hydroxyphenylacetic acid	nd	nd	nd	0.151 ± 0.004 ^a^	1.451 ± 0.060 ^c^	1.402 ± 0.040 ^b^
*p*-hydroxybenzoic acid	nd	nd	nd	0.025 ± 0.001 ^a^	1.495 ± 0.020 ^c^	0.838 ± 0.003 ^b^
Chlorogenic acid	nd	0.038 ± 0.001 ^a^	nd	nd	26.433 ± 1.640 ^c^	15.348 ± 0.850 ^b^
Vanillic acid	0.0650 ± 0.0002 ^a^	nd	nd	nd	0.736 ± 0.040 ^b^	0.749 ± 0.010 ^b^
Caffeic acid	0.0100 ± 0.0003 ^a^	nd	nd	nd	0.093 ± 0.003 ^b^	0.109 ± 0.010 ^c^
Syringic acid	0.0120 ± 0.0002 ^a^	nd	nd	nd	0.169 ± 0.001 ^c^	0.118 ± 0.002 ^b^
*p*-coumaric acid	0.043 ± 0.001 ^bc^	0.122 ± 0.002 ^d^	0.034 ± 0.001 ^ab^	0.044 ± 0.001 ^bc^	3.111 ± 0.010 ^f^	1.333 ± 0.003 ^e^
*o*-vanillin	0.283 ± 0.010 ^c^	0.161 ± 0.003 ^b^	0.066 ± 0.001 ^a^	0.0670 ± 0.0002 ^a^	nd	nd
*o*-coumaric acid	0.0240 ± 0.0001 ^c^	0.014 ± 0.001 ^b^	nd	0.0110 ± 0.0002 ^a^	nd	0.113 ± 0.004 ^d^
Oleuropein	0.403 ± 0.030 ^c^	0.151 ± 0.001 ^b^	0.098 ± 0.002 ^a^	0.106 ± 0.010 ^a^	nd	0.843 ± 0.01 ^d^
Pinoresinol	0.412 ± 0.030 ^d^	0.291 ± 0.002 ^c^	0.146 ± 0.003 ^b^	0.129 ± 0.010 ^a^	nd	nd
Luteolin	nd	0.087 ± 0.003 ^b^	0.070 ± 0.001 ^a^	0.068 ± 0.003 ^a^	nd	nd
Apigenin	0.22800 ± 0.00001 ^b^	0.104 ± 0.001 ^a^	0.121 ± 0.010 ^a^	0.119 ± 0.010 ^a^	1.190 ± 0.010 ^c^	nd
Rutin	nd	nd	nd	nd	nd	nd
**Antioxidant Capacity (µmol TE/g)**						
DPPH	0.883 ± 0.030 ^ab^	0.642 ± 0.070 ^ab^	0.074 ± 0.003 ^a^	0.087 ± 0.002 ^a^	0.877 ± 0.040 ^ab^	0.849 ± 0.010 ^ab^
FRAP	2.451 ± 0.140 ^a^	2.214 ± 0.080 ^a^	0.335 ± 0.010 ^a^	0.247 ± 0.002 ^a^	66.85 ± 2.67 ^b^	99.16 ± 6.11 ^c^
ABTS	2.272 ± 0.190 ^d^	1.524 ± 0.110 ^c^	2.689 ± 0.030 ^e^	0.335 ± 0.020 ^a^	8.976 ± 0.330 ^f^	1.157 ± 0.030 ^b^

Nd = not detected, W/O = Water/oil mixture. ^a–f^ Means with different letters within the same line are significantly different (*p* < 0.05). DPPH: 2,2-diphenyl-1-picrylhydrazyl; FRAP: Ferric reducing ability of plasma; ABTS: 2,2′-azino-bis(3-ethylbenzothiazoline-6-sulphonic acid.

**Table 3:** Phenolic concentration and antioxidant capacity of EVOO and water used during the cooking of tomatoes according to four techniques.

Domestic Cooking Technique	Unused	Deep Fried	Sautéed	Boiled (W/O)	Boiled	Boiled (W/O)
Heat Transfer Medium	Extra Virgin Olive Oil				Water	
**Total Phenol Content (mg GAE/g)**						
Total phenols	0.188 ± 0.010 ^a^	0.209 ± 0.010 ^ab^	0.139 ± 0.002 ^a^	0.039 ± 0.001 ^a^	1.359 ± 0.020 ^c^	3.631 ± 0.210 ^d^
**Individual Phenol Content (µg/g)**						
Gallic acid	nd	Traces	nd	nd	0.194 ± 0.010 ^a^	0.232 ± 0.010 ^b^
Hydroxytyrosol	0.097 ± 0.001 ^b^	1.112 ± 0.010 ^d^	nd	0.088 ± 0.002 ^a^	nd	0.962 ± 0.050 ^c^
3,4-dihydroxybenzoic acid	0.071 ± 0.001 ^a^	0.088 ± 0.003 ^b^	nd	nd	nd	nd
Tyrosol	0.088 ± 0.001 ^a^	0.123 ± 0.003 ^b^	nd	0.085 ± 0.001 ^a^	nd	0.729 ± 0.010 ^c^
*p*-hydroxyphenylacetic acid	nd	0.145 ± 0.001 ^a^	nd	nd	1.374 ± 0.020 ^b^	2.186 ± 0.020 ^c^
*p*-hydroxybenzoic acid	nd	0.024 ± 0.001 ^a^	nd	nd	0.2490 ± 0.0002 ^b^	0.266 ± 0.001 ^c^
Chlorogenic acid	nd	0.048 ± 0.010 ^a^	nd	nd	0.407 ± 0.020 ^b^	0.421 ± 0.010 ^c^
Vanillic acid	nd	nd	nd	0.063 ± 0.001	nd	nd
Caffeic acid	Traces	0.0120 ± 0.0002 ^b^	0.012 ± 0.001 ^ab^	0.010 ± 0.001 ^a^	0.076 ± 0.003 ^d^	0.073 ± 0.001 ^c^
Syringic acid	0.019 ± 0.001 ^c^	0.017 ± 0.001 ^b^	0.0150 ± 0.0001 ^a^	0.019 ± 0.001 ^c^	nd	nd
*p*-coumaric acid	0.041 ± 0.001 ^a^	0.135 ± 0.010 ^b^	nd	0.034 ± 0.002 ^a^	0.418 ± 0.010 ^c^	0.719 ± 0.050 ^d^
*o*-vanillin	0.118 ± 0.010 ^a^	0.176 ± 0.010 ^ab^	0.548 ± 0.630 ^cd^	0.075 ± 0.004 ^a^	0.854 ± 0.020 ^e^	0.691 ± 0.020 ^de^
*o*-coumaric acid	0.015 ± 0.001 ^ab^	0.016 ± 0.001 ^b^	0.011 ± 0.001 ^a^	0.011 ± 0.00003 ^a^	0.101 ± 0.010 ^c^	0.1210 ± 0.0004 ^d^
Oleuropein	0.184 ± 0.010 ^b^	0.193 ± 0.001 ^b^	0.136 ± 0.010 ^a^	0.124 ± 0.010 ^a^	nd	2.127 ± 0.110 ^c^
Pinoresinol	0.234 ± 0.010 ^b^	0.489 ± 0.010 ^d^	0.268 ± 0.020 ^c^	0.186 ± 0.010 ^a^	nd	1.105 ± 0.004 ^e^
Luteolin	nd	0.0800 ± 0.0002 ^c^	0.061 ± 0.001 ^a^	0.072 ± 0.001 ^b^	nd	nd
Apigenin	nd	nd	0.150 ± 0.010 ^b^	0.115 ± 0.010 ^a^	1.127 ± 0.010 ^d^	1.039 ± 0.050 ^c^
Rutin	nd	nd	nd	nd	1.035 ± 0.050	nd
**Antioxidant Capacity (µmol TE/g)**						
DPPH	1.446 ± 0.002 ^b^	2.912 ± 0.320 ^c^	1.456 ± 0.110 ^b^	0.359 ± 0.010 ^a^	0.516 ± 0.020 ^a^	1.418 ± 0.080 ^b^
FRAP	0.851 ± 0.010 ^c^	2.307 ± 0.130 ^e^	1.389 ± 0.090 ^d^	0.411 ± 0.030 ^ab^	0.149 ± 0.001 ^a^	0.525 ± 0.050 ^bc^
ABTS	0.523 ± 0.020 ^cd^	0.855 ± 0.090 ^e^	0.5990 ± 0.0004 ^d^	0.155 ± 0.010 ^a^	0.291 ± 0.010 ^b^	0.544 ± 0.020 ^cd^

Nd = not detected, W/O = Water/oil mixture. ^a–e^ Means with different letters within the same line are significantly different (*p* < 0.05). DPPH: 2,2-diphenyl-1-picrylhydrazyl; FRAP: Ferric reducing ability of plasma; ABTS: 2,2′-azino-bis(3-ethylbenzothiazoline-6-sulphonic acid.

**Table 4:** Phenolic concentration and antioxidant capacity of EVOO and water used during the cooking of pumpkins according to four techniques.

Domestic cooking technique	Unused	Deep Fried	Sautéed	Boiled (W/O)	Boiled	Boiled (W/O)
Heat Transfer Medium	Extra Virgin Olive Oil				Water	
**Total Phenol Content (mg GAE/g)**						
Total phenols	0.37 ± 0.01 ^e^	0.2990 ± 0.0001 ^d^	0.146 ± 0.010 ^c^	0.062 ± 0.001 ^b^	0.0350 ± 0.0003 ^a^	0.072 ± 0.001 ^b^
**Individual Phenol Content (µg/g)**						
Gallic acid	nd	nd	nd	nd	nd	nd
Hydroxytyrosol	0.196 ± 0.010 ^b^	0.142 ± 0.010 ^ab^	0.085 ± 0.001 ^a^	nd	nd	4.677 ± 0.090 ^c^
3,4-dihydroxybenzoic acid	nd	nd	nd	nd	nd	0.708 ± 0.012
Tyrosol	0.154 ± 0.011 ^c^	0.129 ± 0.010 ^b^	0.072 ± 0.004 ^a^	nd	nd	0.727 ± 0.012 ^d^
*p*-hydroxyphenylacetic acid	nd	nd	nd	nd	1.720 ± 0.066	nd
*p*-hydroxybenzoic acid	nd	nd	nd	nd	0.377 ± 0.010 ^b^	0.283 ± 0.020 ^a^
Chlorogenic acid	nd	nd	nd	nd	0.38 ± 0.02 ^a^	0.444 ± 0.030 ^b^
Vanillic acid	0.0650 ± 0.0004 ^a^	0.065 ± 0.001 ^a^	nd	nd	nd	0.685 ± 0.030 ^b^
Caffeic acid	0.0120 ± 0.0002 ^a^	0.018 ± 0.001 ^b^	Traces	nd	0.073 ± 0.001 ^c^	0.084 ± 0.001 ^d^
Syringic acid	0.0130 ± 0.0003 ^a^	0.0130 ± 0.0003 ^a^	0.012 ± 0.001 ^a^	0.013 ± 0.001 ^a^	0.117 ± 0.010 ^b^	0.124 ± 0.004 ^c^
*p*-coumaric acid	nd	0.096 ± 0.010 ^c^	0.080 ± 0.002 ^b^	0.045 ± 0.001 ^a^	nd	0.912 ± 0.001 ^d^
*o*-vanillin	0.267 ± 0.010 ^d^	0.252 ± 0.010 ^c^	0.096 ± 0.010 ^b^	0.076 ± 0.003 ^a^	0.745 ± 0.010 ^e^	0.783 ± 0.040 ^f^
*o*-coumaric acid	0.035 ± 0.002 ^b^	0.023 ± 0.001 ^ab^	0.014 ± 0.001 ^a^	0.012 ± 0.001 ^a^	0.494 ± 0.030 ^d^	0.254 ± 0.004 ^c^
Oleuropein	0.794 ± 0.014 ^c^	0.144 ± 0.003 ^a^	0.221 ± 0.001 ^b^	0.128 ± 0.010 ^a^	nd	1.463 ± 0.090 ^d^
Pinoresinol	0.538 ± 0.020 ^d^	0.459 ± 0.020 ^c^	0.292 ± 0.010 ^b^	0.175 ± 0.010 ^a^	nd	1.084 ± 0.040 ^e^
Luteolin	0.123 ± 0.002 ^d^	0.114 ± 0.004 ^c^	0.089 ± 0.010 ^b^	0.076 ± 0.010 ^a^	nd	nd
Apigenin	nd	0.101 ± 0.010	nd	nd	nd	nd
Rutin	nd	nd	nd	nd	nd	nd
**Antioxidant Capacity (µmol TE/g)**						
DPPH	0.908 ± 0.010 ^f^	0.686 ± 0.030 ^e^	0.309 ± 0.020 ^d^	0.112 ± 0.010 ^b^	0.006 ± 0.001 ^a^	0.204 ± 0.010 ^c^
FRAP	2.436 ± 0.080 ^d^	2.836 ± 0.690 ^e^	1.552 ± 0.060 ^c^	0.415 ± 0.200 ^b^	0.034 ± 0.0004 ^a^	0.468 ± 0.020 ^b^
ABTS	1.313 ± 0.020 ^d^	1.175 ± 0.020 ^c^	0.719 ± 0.050 ^b^	0.323 ± 0.010 ^a^	0.252 ± 0.010 ^a^	0.761 ±0.100 ^b^

nd = not detected, W/O = Water/oil mixture. ^a–f^ Means with different letters within the same line are significantly different (*p* < 0.05). DPPH: 2,2-diphenyl-1-picrylhydrazyl; FRAP: Ferric reducing ability of plasma; ABTS: 2,2′-azino-bis(3-ethylbenzothiazoline-6-sulphonic acid.
